# Biological control of the cucurbit powdery mildew pathogen *Podosphaera xanthii* by means of the epiphytic fungus *Pseudozyma aphidis* and parasitism as a mode of action

**DOI:** 10.3389/fpls.2015.00132

**Published:** 2015-03-11

**Authors:** Aviva Gafni, Claudia E. Calderon, Raviv Harris, Kobi Buxdorf, Avis Dafa-Berger, Einat Zeilinger-Reichert, Maggie Levy

**Affiliations:** ^1^Plant Pathology and Microbiology, Hebrew University of JerusalemJerusalem, Israel; ^2^The Interdepartmental Equipment Facility, The Robert H. Smith Faculty of Agriculture, Food and Environment, Hebrew University of JerusalemJerusalem, Israel

**Keywords:** dimorphism, biocontrol, powdery mildew, phytopathogens, parasitism

## Abstract

Epiphytic yeasts, which colonize plant surfaces, may possess activity that can be harnessed to help plants defend themselves against various pathogens. Due to their unique characteristics, epiphytic yeasts belonging to the genus *Pseudozyma* hold great potential for use as biocontrol agents. We identified a unique, biologically active isolate of the epiphytic yeast *Pseudozyma aphidis* that is capable of inhibiting *Botrytis cinerea* via a dual mode of action, namely induced resistance and antibiosis. Here, we show that strain L12 of *P. aphidis* can reduce the severity of powdery mildew caused by *Podosphaera xanthii* on cucumber plants with an efficacy of 75%. Confocal and scanning electron microscopy analyses demonstrated *P. aphidis* proliferation on infected tissue and its production of long hyphae that parasitize the powdery mildew hyphae and spores as an ectoparasite. We also show that crude extract of *P. aphidis* metabolites can inhibit *P. xanthii* spore germination *in planta*. Our results suggest that in addition to its antibiosis as mode of action, *P. aphidis* may also act as an ectoparasite on *P. xanthii*. These results indicate that *P. aphidis* strain L12 has the potential to control powdery mildew.

## Introduction

Plant pathogens challenge our efforts to maximize crop production. Fungi, bacteria and other pathogens attack various parts of crop plants, inducing diseases that reduce the plant's growth rate, suppress plant development and reduce yield. Agronomists use chemical-based pesticides to control the spread of pathogens and pests and help ensure stable and prosperous agricultural systems. Nevertheless, constant selective pressures from multiple applications has led to the development of pathogens and pests that are resistant to chemical pesticides and consequently efforts are being made to develop novel techniques and tools to control these pests and pathogens (Denholm and Rowland, 1992; Leroux et al., 2002). Among these tools, fungal biocontrol agents have attracted much attention as a viable and important alternative to conventional pesticides. Biological control may involve one or a combination of mechanisms, such as antibiosis, mycoparasitism, competition and the induction of generalized resistance in the host plant (Elad and Freeman, 2002; Shoresh et al., 2010). These mechanisms can hinder pathogen growth and development, thereby reducing disease. The complex modes of action of biocontrol agents, unlike those of chemical agents, reduce the likelihood of pathogens developing resistance to these agents, which increases the appeal of biocontrol agents as an alternative to conventional chemical products in the battle against pests and pathogens.

Epiphytic yeasts that colonize different plant surfaces (Pusey et al., 2009; Fernandez et al., 2012) are thought to possess biocontrol activity and to provide a natural barrier against certain plant pathogens (Avis et al., 2001; Urquhart and Punja, 2002; Bleve et al., 2006; Jacobsen, 2006; Robiglio et al., 2011). Epiphytes usually produce extracellular polysaccharides, which are thought to help them survive on aerial plant surfaces that are exposed to the elements. Phylloplane yeasts can also metabolize a wide variety of nutrient sources and tolerate a variety of chemical-based fungicides (Barnett et al., 2000; Buck and Burpee, 2002), characteristics which could potentially contribute to their utility as biocontrol agents.

The genus *Pseudozyma* is a small group of yeast-like fungi classified among the *Ustilaginales* (Boekhout, 1995). They are mostly epiphytic or saprophytic and they are non-pathogenic to plants, animals and insects (Avis and Belanger, 2002). *Pseudozyma rugulosa* and *Pseudozyma flocculosa* have both been reported to exhibit biological activity against the different powdery mildews with which they are associated (Dik et al., 1998; Hammami et al., 2010, 2011) (for review see Kiss, 2003). *P. flocculosa*, for example, does not penetrate powdery mildew cells, but has been found to secrete an unusual fatty acid that has an antibiotic effect against powdery mildew and other pathogens (Hajlaoui et al., 1994; Benyagoub et al., 1996; Avis and Belanger, 2001; Avis et al., 2001). Recently the genome and transcriptome of the biocontrol agent *P. flocculosa* was published and compared to the related plant pathogen *Ustilago maydis* and other Ustilaginales pathogens (Lefebvre et al., 2013). Genome comparison demonstrated high similarity of genomes, including the mating-type loci, meiosis loci and pathogenicity components, suggesting *P. flocculosa* used to be a virulent smut pathogen. Furthermore, it was shown that *P. flocculosa* has lost a subset of secreted effectors reported to influence virulence while acquiring other genes coding for secreted proteins, not found in the pathogenic fungi, that probably contribute to its biocontrol nature (Lefebvre et al., 2013). In agreement with these findings, the genome and transcriptome of *P. antarctica*, that did not demonstrate biocontrol ability against powdery mildew (Avis et al., 2001), shares a high degree of syntany to the pathogenic fungus *U. maydis*. However, the transcriptome analysis reveals significant differences regarding pathogenicity and metabolism, indicating *P. antarctica* has an oleaginous nature which is relevant to its non-pathogenic characteristics (Morita et al., 2013b, 2014).

*Pseudozyma aphidis* is a close relative of the powdery mildew biocontrol agent *P. rugulosa* (Begerow et al., 2000). *P. aphidis* was first isolated from aphid secretions (Henninger and Windisch, 1975) and later identified on plant surfaces as well (Allen et al., 2004). Recently the sequence of *P. aphidis* isolate DSM70725 that was isolated from aphid secretions was published (Lorenz et al., 2014). Previous studies on another *P. aphidis* strain that was also isolated from aphid secretions (isolate CBS517.83) indicated that this species does not produce any unique fungitoxic fatty acids (Avis and Belanger, 2001) and is not associated with the collapse of powdery mildew colonies [*Podosphaera xanthii* formerly *Sphaerotheca fuliginea* (Schlechtend.:Fr.) Pollacci].

We recently reported on a unique active isolate of *P. aphidis* that was identified on the surface of strawberry leaves in association with powdery mildew collapse (designated isolate L12). We demonstrated this isolate's activity against *Botrytis cinerea* colonization and spread on tomato (*Solanum lycopersicum*) and *Arabidopsis thaliana* plants. That observed biocontrol effect was based on a dual mode of action: antibiosis and induced resistance (Buxdorf et al., 2013a,b). The induced resistance was found to be both SA/NPR1- and JAR1/EIN2-independent (Buxdorf et al., 2013a,b).

Powdery mildew is the most common disease of cucurbits and a serious threat in many countries. *P. xanthii* is considered to be the main causal agent of powdery mildew on cucurbits, one of the most important limiting factors for cucurbit production (Bélanger et al., 2002; Perez-Garcia et al., 2009). The constant fungicide application in the field to control this pathogen has led to the development of resistance to various chemicals, thereby reducing the effectiveness of these treatments (McGrath, 2001; Hollomon et al., 2002). Thus, efforts are being made to develop novel techniques to control powdery mildew in the field.

Here, we present a potentially efficient biological control agent against cucurbit powdery mildew that relies on the biologically active *P. aphidis* strain (isolate L12). We demonstrate in the current work the ability of this isolate to antagonized powdery mildew on cucumber plants, using parasitism and antibiosis as modes of action.

## Materials and methods

### Propagation of *P. aphidis* and pathogens

*Pseudozyma aphidis* strain L12 was maintained on potato dextrose agar (PDA; Difco, France) at 25°C. An indigenous population of *Podosphaera xanthii* was maintained on squash (*Cucurbita maxima*) plants under field conditions.

### Propagation of plants

Cucumber (*Cucumis sativus* cv. Saphi) plants were grown at 25°C under 40% relative humidity in the greenhouse.

### Fluorescence microscopy

A GFP-labeled *P. aphidis* (see Supplementary Figure 1 and Supplementary Methods) was used to visualize colonization of healthy and infected tissue. Ten-day-old cucumber cotyledons were sprayed with either water or GFP-*P. aphidis* (10^8^ cells/ml of sterile, deionized water). Three days after treatment the cotyledons were placed on tap water agar medium and then inoculated with *P. xanthii*. Inoculation with *P. xanthii* was done by brushing cucumber cotyledons at several points with a brush that had been vigorously rubbed on squash (*Cucurbita maxima*) leaves that had been intensely colonized by *P. xanthii*.

The inoculated cucumber cotyledons were layered on agar trays and placed in a controlled-environment chamber where they were kept at 22°C at 80–90% relative humidity and under fluorescent and incandescent light with a photofluency rate of approximately 120 μmol/m^2^s and a 12-h photoperiod. Two–seven days after inoculation with *P. xanthii*, the cotyledons were examined with a stereomicroscope (Nikon SMZ 1500) with an epifluorescence attachment (Nikon P-FLA-2). The intensity of the green fluorescence produced by excitation at 488 nm was measured and a GFP filter was used for visualization (Nikon, GFP-B). Images were taken using a Nikon Ds-Ri1camera and processed with NIS elements BR 3.10 software. Cotyledons were also examined with confocal laser scanning microscope (TCS SP8, Leica) 7 days after inoculation with *P. xanthii*; GFP was excited using a 488 nm laser and maximum emission was set at 500 nm. Cucumber cotyledons were also treated with 1mg/ml propidium iodide that was excited using a 514 nm laser and maximum emission was set at 610 nm. The images were analyzed in the LAS AF (Leica) computer program, at a resolution of 1024 × 1024 pixels. The experiments were repeated three times.

### Inhibition of powdery mildew on cucumber seedlings

Two-week-old greenhouse-grown (25°C and 40% relative humidity) cucumber seedlings (with two true leaves) were sprayed with *P. aphidis* cells suspended in water (10^8^ cells/ml) or water (*n* = 10) 3 days before they were inoculated with *P. xanthii*. Inoculation with *P. xanthii* was performed by brushing cucumber leaves with a brush that had been vigorously rubbed on squash (*Cucurbita maxima*) leaves that were intensely colonized by *P. xanthii*. Infection severity was scored 11, 12, and 16 days post-inoculation by evaluating the percentage of the infected area on each of the first two leaves. The experiment was concluded after 16 days when the first leaf of the control treatment was fully coved with powdery mildew. Percentage of powdery mildew coverage (PMC) was estimate and disease severity was scored using the 12-grade scale described by Horsfall and Barratt (1945) with minor modifications: 0 = 0%, 1 = 0–3%, 2 = 3–6%, 3 = 6–12%, 4 = 12–25%, 5 = 25–50%, 6 = 50–75%, 7 = 75–87%, 8 = 87–94%, 9 = 94–97%, 10 = 97–100%, 11 = 100% disease. A mean disease-severity index value (DSI) was calculated for each treatment by adding up the scores of the 20 leaves in the treatment and then expressing that sum as a percentage using the formula described by Raupach et al. (1996): Disease Severity Index = [∑(rating no. × no. of plants in rating) × 100%]/(total no. of plants × highest rating). Biocontrol efficacy was calculated as: Efficacy = [(disease rate in control − disease rate in treatment)/disease rate in control] × 100. The entire experiment was performed three times with similar results.

### Scanning electron microscopy

For scanning electron microscopy (SEM), *P. aphidis*-treated and powdery mildew-inoculated cucumber cotyledons were collected 1–4 days after inoculation and fixed with glutaraldehyde using standard protocols (Weigel and Glazebrook, 2002). Samples were mounted on aluminum stubs and sputter-coated with AU-Pd. SEM was performed using a Jeol JEM 5410 at 20 kV.

### Transmission electron microscopy

For transmission electron microscopy (TEM), cucumber cotyledons were treated with *P. aphidis* inoculated with *P. xanthii* 3 days later, and collected 8 days post-inoculation and processed using a standard protocol (Chuartzman et al., 2008). Epon-embedded samples were sectioned using a diamond knife on an LKB 3 microtome (Leica, Bensheim, Germany) and ultrathin sections (80 nm) were collected onto 200 Mesh, thin bar copper grids. The sections on grids were sequentially stained with Uranyl acetate and Lead citrate for 10 min each and observed with a Technai T12 TEM 100 kV (Phillips, Eindhoven, the Netherlands).

### Antibiosis assays using crude extract of *P. aphidis* metabolites

Metabolites were extracted from *P. aphidis* cells using ethyl acetate. One and a half liters of PDB medium in a 3-l Erlenmeyer flask were inoculated with two 1-cm^2^ blocks of PDA carrying mycelia and/or spores of *P. aphidis* and grown for 10 days at 27°C, in dark with constant agitation at 150 rpm. We then spun down the fungal cells (5 min at 7000 rpm) and extracted them with a 3-l of ethyl-acetate. The ethyl-acetate fraction was collected and evaporated in a rotor evaporator (Buchi, Flawil, Switzerland) at 42°C (Paz et al., 2007). Evaluation of the inhibitory effect of the metabolites extracted from *P. aphidis* on *P. xanthii* conidia germination was carried out using cucumber cotyledons from 1-week-old plants. Cotyledons disks (15 mm in diameter) were disinfected with 0.1% NaOCl (w/v) for 2 min, and rinsed twice in sterile distilled water. The crude extract (200 mg/ml in ethyl-acetate) was diluted with distilled water (1:15 v/v) and poured into sterile six-well plates. Distilled water and an ethyl acetate diluted in distilled water (1:15 v/v) were used as controls. The cotyledon disks were placed in these dilutions upside down and incubated at room temperature for 10 min. After incubation, the disks were transferred onto solid medium (40 g sucrose, 30 mg benzimidazole, 10 g agar, 1-l distilled water) in 5 cm diameter Petri dishes, and the upper side of the disks was inoculated with conidia of *P. xanthii* with a brush. After 24 h of incubation (22°C and 16 h of daylight), disks were cleared in boiling absolute ethanol for 20 min in a water bath, followed by a final wash in glycerol: lactic acid: water (1:1:1 v/v) overnight. The disks were then incubated for 2 min in aniline blue (0.2%) followed by rinsing in distilled water, and then examined under a bright-field microscope. The percentage of spore-inhibition was calculated in relation to untreated control with distilled water. Spore germination in water ranged between 98 and 100%. For each experiment the means of the distilled water control values were considered as a 100% germination; all other values were divided by these values and multiplied by a 100 for obtaining the percentage of spore germination. To obtain percentage of spore inhibition the percentage of spore germination was reduced from a 100.

### Chitinase activity

To evaluate chitinase activity, *P. aphidis* was grown on tap water agar plates (TWA; 2% agar) or TWA supplemented with 0.1% (w/v) chitin (Sigma-Aldrich, USA). Colony diameter was measured 8 and 17 days post-inoculation.

### Data analysis

Student's *t*-test was performed only when data were normally distributed and the sample variances were equal. Significance was accepted at *P* < 0.05, as noted in the text or table captions. All experiments described here are representative of at least two independent experiments with the same pattern of results.

## Results

### Antagonistic effect of *P. aphidis* against powdery mildew *in planta*

**Figure 1:**
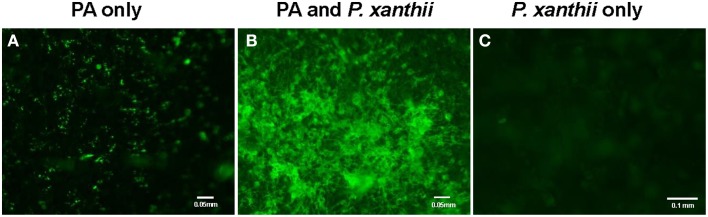
**Imaging of *P. aphidis* proliferation on infected cucumber cotyledons**. Fluorescence microscopy analysis of cucumber cotyledons treated with GFP-tagged *P. aphidis* **(A)** followed by successive inoculation (3 days after *P. aphidis* treatment) with *P. xanthii* **(B)** or treated with water and inoculated with *P. xanthii* **(C)**. Images were recorded 5 days after inoculation *P. xanthii*. Shown are representative pictures taken of one cotyledon out of 5 from each treatment in one representative experiment. The entire experiment was performed three times with similar results.

We first demonstrated the ability of *P. aphidis* to maintain a stable population on cucumber (*C. sativus*) seedlings. Specifically, we treated 10-day-old seedlings with a *P. aphidis* suspension and monitored the number of CFU appearing over a period of 21 days post-inoculation. The size of the *P. aphidis* remained stable for 18-days on plants maintained under controlled conditions (Supplementary Figure 2). Foliar application of *P. aphidis* (10^8^ cells/ml) to cucumber plants did not result in any symptoms of pathogenicity or phytotoxicity, such as chlorosis. Furthermore, following application of GFP-expressing *P. aphidis* (Supplementary Figure 1) to healthy cucumber cotyledons, we observed minor spreading of the green fluorescence cells throughout the cotyledons (Figure 1A). When *P. aphidis*-treated cucumber cotyledons were then inoculated with *P. xanthii*, a stronger, denser and more uniform green fluorescent signal was observed in the inoculated area (Figure 1B), indicating the close association of *P. aphidis* with *P. xanthii*.

We then tested the ability of *P. aphidis* to control cucumber powdery mildew *in vivo*. Greenhouse-grown cucumber seedlings (two leaves) were sprayed with *P. aphidis* cells or with water as a control and 3 days later, seedlings were inoculated with *P. xanthii*. The application of *P. aphidis* significantly reduced the severity of powdery mildew disease symptoms on the treated cucumber plants as compared with control untreated infected plants (Table 1). Sixteen days after inoculation, almost all first two leaves of the untreated plants showed close to 100% coverage with powdery mildews, compared to only 17% coverage on the treated plants (Table 1 and Supplementary Figure 3). Moreover, the appearance of disease symptoms was delayed by 12 days on the treated plants as compared to the untreated ones. After 11 days, there was 45% powdery mildew coverage of the untreated leaves, whereas all of the treated leaves were still symptomless (Table 1). Disease severity 12 days post-inoculation was 15% for plants treated with *P. aphidis* as compared to 88% for control plants, and 16 days post-inoculation, 31% for *P. aphidis*-treated plants vs. 98% for untreated control plants (Table 1). The efficacy of *P. aphidis* treatment was 75% on day 16 post-inoculation (Table 1).

**Table 1:** **Effect of *P. aphidis* treatment on severity of disease symptoms caused by *P. xanthii***.

**Treatment**	**DSI (%)**			**PMC (%)**			**Efficacy (%)**		
	**11 dpi**	**12 dpi**	**16 dpi**	**11 dpi**	**12 dpi**	**16 dpi**	**11 dpi**	**12 dpi**	**16 dpi**
PA-treated	0	15	31	0^*^	3.2 ± 1.7^*^	16.9 ± 9.1^*^	100	88	75
Control	67	88	98	46.5 ± 4.7	56.5 ± 4.7	98.7 ± 2.5			

*Disease severity index (DSI) and P. xanthii average leaf coverage (PMC) were calculated 11, 12 and 16 days post-inoculation (dpi) as described by Raupach et al. (1996). PA, P. aphidis. Means and standard errors of PMC are presented. Means of DSI followed by asterisks is significantly different from inoculated water-treated control according to Student's t-test; P < 0.05; n = 20 leaves from 10 different plants*.

### Morphological yeast-to-hypha transition and ectoparasitism of *P. aphidis*

**Figure 2:**
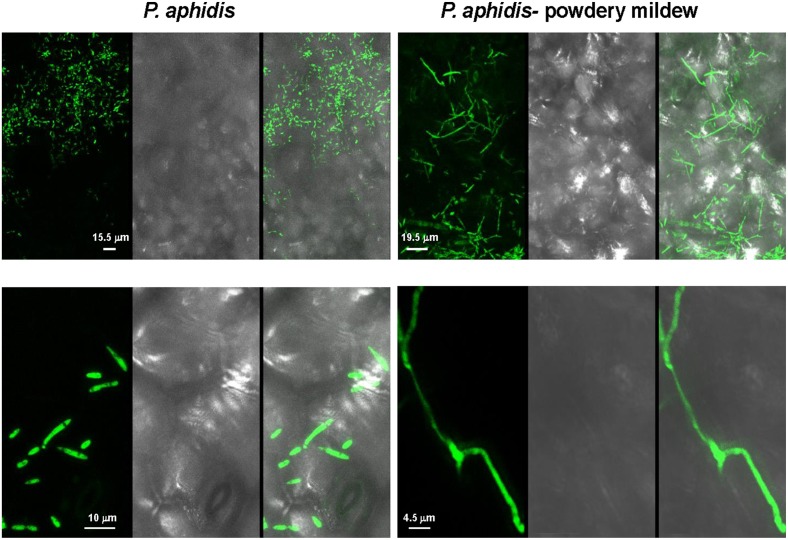
***P. aphidis* forms long hyphae when interacting with powdery mildew**. Confocal microscopy observations of cucumber cotyledons treated with GFP-tagged *P. aphidis* (PA-GFP). PA-GFP-treated cotyledons 4 days after inoculation with *Podosphaera xanthii* (*P. aphidis*-powdery mildew) or water (*P. aphidis*). The panels, from left to right, depict fluorescence, white light, and overlay images, respectively, for each of the treatments.

**Figure 3:**
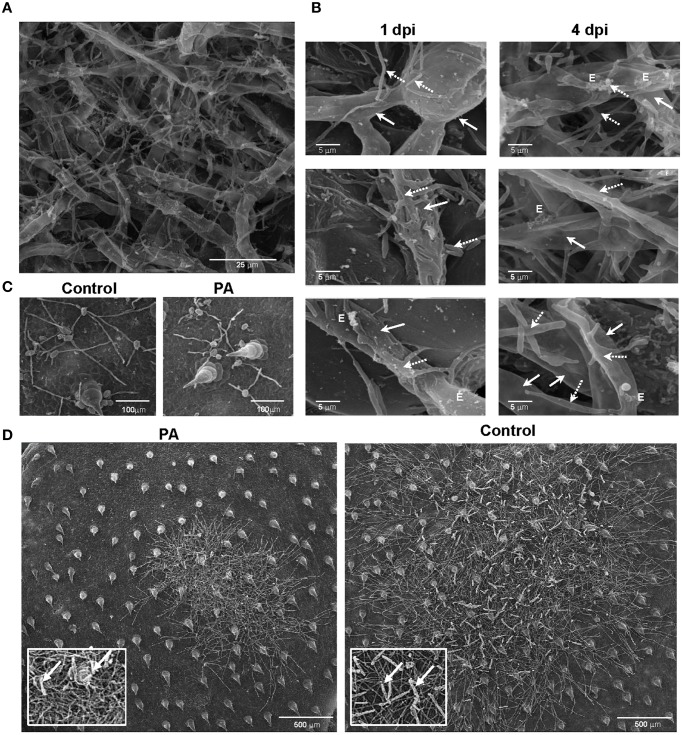
***P. aphidis*–powdery mildew interactions on cucumber cotyledons**. SEM microscopy of cucumber cotyledons treated with *P. aphidis* and infected with *Podosphaera xanthii*. **(A)** Cucumber cotyledons treated with *P. aphidis* 4 days post-infection with *P. xanthii*. **(B)** Closer look at the interaction of *P. aphidis* and *P. xanthii* mycelium and spores 1 and 4 days post-infection with *P. xanthii*. Cucumber cotyledons treated with *P. aphidis* (PA) or with water (Control) 1 day post-inoculation **(C)** and 10 days post-inoculation with *P. xanthii* **(D)** (sporulation marked with white arrows). **(A–C)** *P. xanthii* mycelium and spores are indicated with white arrows and *P. aphidis* cells and hyphae with dashed arrows; E, extracellular matrix.

**Figure 4:**
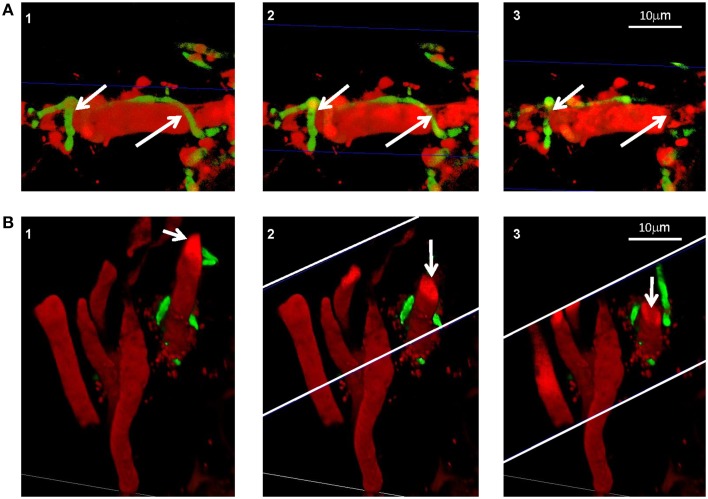
***P. aphidis* as an ectoparasite**. Confocal microscopic analysis of cucumber cotyledons treated with propidium iodide 10 days after treatment with GFP-tagged *P. aphidis* (green) and 7 days after inoculation with *P. xanthii* (red). **(A)** Reconstructed 3D images demonstrating *P. aphidis* (green; marked with white arrows) coiling around the powdery mildew hyphae and cross-sections (marked with blue thin lines) demonstrating no fluorescence inside the powdery mildew hypha. See Supplemental Movie 1 for whole series of sections. **(B)** Reconstructed 3D cross-section images. The white thin lines marked the area of the cross-sections, while the white arrows point to the sectioned powdery mildew hypha. See Supplemental Movie 2 for whole series of sections.

**Figure 5:**
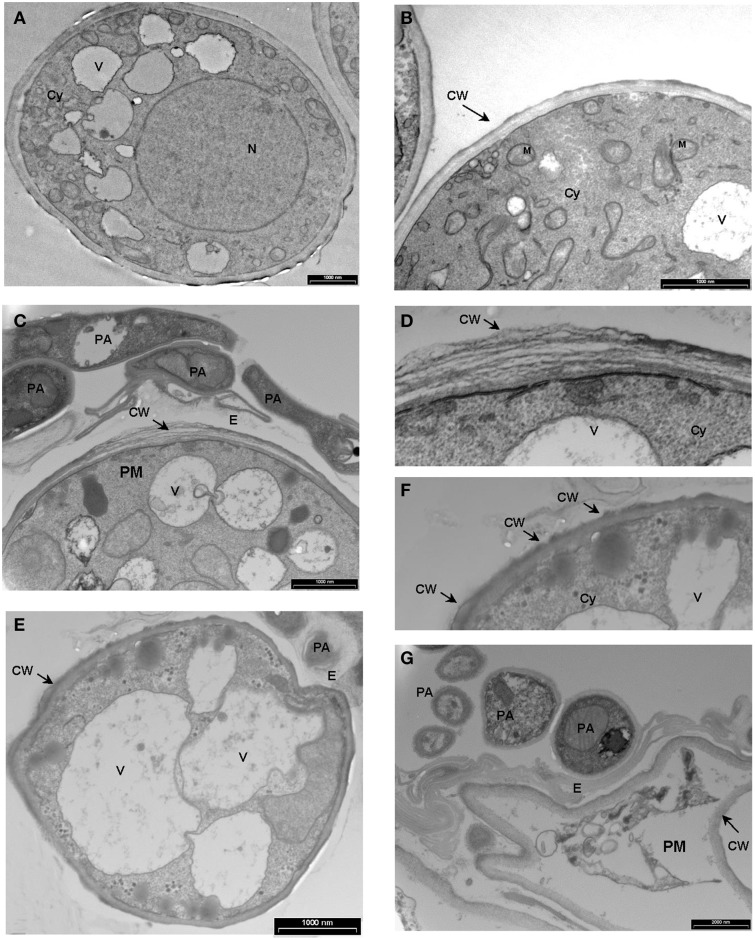
**Transmission electron micrographs depicting the interaction between *P. Xanthii* and *P. aphidis* on cucumber leaves**. **(A,B)** Ultrastructure of *P. xanthii* (PM) hypha on cucumber leaves 8 days post-inoculation. Hyphal cells are surrounded by an intact cell wall (CW) and show a dense polyribosome—rich cytoplasm (Cy), with numerous organelles inside, including mitochondria (M), nucleus (N) and vacuoles (V). **(C–G)** Ultrastructure of *P. xanthii* and *P. aphidis* (PA) interactions on cucumber leaves 8 days post-inoculation and 11 days post-treatment with *P. aphidis*. **(C)** *P. aphidis* cells encircle the *P. xanthii* hyphae causing deformation of the cell wall (CW), also seen at higher magnification in **(D)**. **(E)** Increased vacuolation is accompanied by deformation of the cell wall and disorganization of the cytoplasm, also seen at higher magnification in **(F)**. **(G)** *P. xanthii* cells are markedly collapsed. E, extracellular matrix.

We demonstrated *in vitro* that *P. aphidis* is a dimorphic yeast-like fungus that grows mainly as a yeast in PDB medium but can form hyphae when grown in different media, such as YMPD or MS (Supplementary Figure 4). Using confocal microscopy, we demonstrated the morphological yeast-to-hypha transition (dimorphism) of *P. aphidis in planta* (Figure 2). *P. aphidis* was mainly in yeast form on uninfected tissue (Figure 2 Left), whereas on *P. xanthii*-infected tissue, it was mainly in hyphal form (Figure 2 Right). SEM revealed that the areas infected with powdery mildew were covered with *P. aphidis* (Figure 3), and that the powdery mildew's hyphae were significantly shorter (by 27%, Supplementary Table 1) when they were associated with *P. aphidis* at infection initiation 2 dpi (Figure 3C). *P. aphidis*, on the other hand, formed long hyphae that branched when associated with *P. xanthii* and attached to the powdery mildew hyphae by coiling around them as seen by SEM analysis (Figures 3A,B). We observed inhibition of growth and sporulation of powdery mildew on the plants that were treated with *P. aphidis* as compared to control (Figure 3D). We also observed accumulation of extracellular matrix in the area of interaction between *P. aphidis* and powdery mildew, and that the hyphae of *P. aphidis* extending from one powdery mildew hypha to another one were thinner (Figures 3A,B and Supplementary Figure 5). Furthermore, *P. aphidis* behave like an ectoparasite, as demonstrated by confocal and TEM microscopy. We could not detect any GFP fluorescence or *P. aphidis* cells inside the powdery mildew hyphae using confocal microscopy or TEM, respectively (Figures 4, 5 and Supplementary Movies 1, 2). TEM analysis of powdery mildew cells associated with *P. aphidis* showed numerous abnormalities: increased vacuolation, deformation of the cell wall and disorganization of the cytoplasm. These abnormalities eventually lead to collapse of the powdery mildew cells (Figure 5). Accumulation of extracellular matrix surrounding *P. aphidis* was also observed (Figure 5).

### *P. aphidis* metabolites inhibit *P. xanthii* spore germination *in planta* and poses chitinase activity

While the precise biochemical nature of the pinkish substance secreted by *P. aphidis* is still unknown, we hypothesized that *P. aphidis* secretes antibiotics and enzymes, such as chitinase, that can inhibit and degrade fungal cell walls. For antibiosis activity, we used *P. aphidis* extract and revealed that crude extract of *P. aphidis* metabolites can almost completely inhibit *P. xanthii* spore germination on intact cucumber cotyledons (Table 2). We also demonstrated that the vast majority of spores that are germinated on extract form a one germination tube and not hypha (Supplementary Figure 6).

**Table 2:** **Inhibition of spore germination by. *P. aphidis* crude extract**.

**Treatment**	**Inhibition of spore germination (%)**
PA	97.5 ± 1.7^*^
Control	2.3 ± 4.3

*Percentage of spore inhibition after 24 h on cucumber cotyledon disks covered with P. aphidis crude extract (PA) or EtOAc (Control). Data represent the means ± standard deviation. Value followed by an asterisk is significantly different from the control according to Student's t-test; P < 0.001; n = 7 replicates per treatment with 100 spores each*.

**Figure 6:**
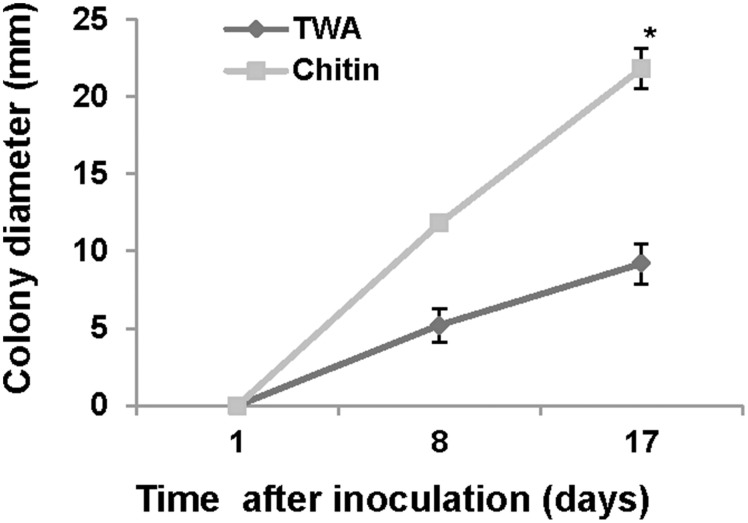
**Chitinase activity**. Growth of *P. aphidis* on 0.1% chitin as compared to TWA. Averages of five samples are presented with standard-error bars. Asterisks indicate a significant difference as determined by Student's *t*-test; *P* < 0.05.

For chitinase activity verification we grew *P. aphidis* on TWA plates supplemented with chitin as sole carbon source. On TWA plates supplemented with chitin, *P. aphidis* colony diameter was 21.8 mm as compared to 9.1 mm on TWA alone (Figure 6). These findings suggest that *P. aphidis* can secrete chitinase to utilize chitin as a carbon source.

## Discussion

Fungal biocontrol agents are important for disease control and can provide a viable alternative for chemical-based pesticides. Yet the number of fungal biocontrol agents that are currently used for practical applications is minuscule as compared with the use of chemical-based agents. We recently identified a unique isolate of *P. aphidis* (isolate L12) from strawberry leaves associated with powdery mildew collapse and demonstrated that this isolate can reduce *B. cinerea* infection of tomato and *Arabidopsis* plants (Buxdorf et al., 2013a,b). That biocontrol effect against *B. cinerea* was found to proceed via a dual mode of action, antibiosis and induced resistance that is SA/NPR1- and JA/ET-independent (Buxdorf et al., 2013a,b). Here, we studied the ability of this isolate to control the growth of another pathogen. We demonstrated that *P. aphidis* can colonize healthy plant leaf surfaces and that it can proliferate on powdery mildew infected cucumber cotyledons (Figures 1–3, and Supplementary Figure 2), as was also demonstrated for its close relative *Pseudozyma flocculosa*, which can colonize cucumber leaves that have been inoculated with powdery mildew (Hammami et al., 2011).

The *P. aphidis* isolate studied in this work was found to control powdery mildew (Table 1) via parasitism (Figures 3–5) and antibiosis (Table 2). This is in contrast to the findings reported in previous studies by Avis et al. (2001). The difference between our findings and those of Avis et al. (2001) is likely due to the different isolates used. It is particularly noteworthy that the application of *P. aphidis* prior to pathogen inoculation significantly reduced the severity of powdery mildew on greenhouse-grown cucumber plants with an efficacy of 75% (Table 1). We and others have previously reported that applying a biocontrol agent before pathogen infection can indeed improve biocontrol activity (Filonow et al., 1996; Buxdorf et al., 2013a,b), but other reports have suggested otherwise (Chalutz and Wilson, 1990; Cook et al., 1997).

We demonstrated that *P. aphidis* occupies the same niches as powdery mildew (Figures 1–3), as also observed previously with *B. cinerea* (Buxdorf et al., 2013b) and similar to observations made with its close relative the biocontrol agent *P. flocculosa* in association with powdery mildew (Hammami et al., 2011). *P. aphidis* isolate L12 was also shown to have a dimorphic morphology, forming yeast-like structures and short pseudo-hyphae on plant surfaces or when interacting with *B. cinerea* (Buxdorf et al., 2013b). Here we demonstrated that *P. aphidis* mainly forms yeast-like structures on uninfected leaves and hyphae on infected tissue that lengthen with infection development (Figures 2, 3). Similarly, Hammami et al. (2010, 2011) demonstrated pseudo-hyphal growth of *P. flocculosa* under stress conditions as compared to yeast-like growth under control conditions (Hammami et al., 2010, 2011). In many other dimorphic fungi, the morphological choice of yeast or mycelium is associated with quorum sensing, inoculum size, stress conditions and secreted molecules (Hornby et al., 2001, 2004; Hogan, 2006; Nickerson et al., 2006; Berrocal et al., 2014). Since we did not observed coiling or the formation of long hyphae when *P. aphidis* interacted with *B. cinerea*, we assume that the interaction with powdery mildew triggers this yeast–mycelia transition, probably involving molecule secretion or other cues that activate gene expression and need to be further characterized. We demonstrated that *P. aphidis* attaches by coiling around the powdery mildew hyphae, forming a hyphal network. While we could observe strong attachments of *P. aphidis* to the powdery mildew hyphae and appressorium-like structures using SEM (Figure 3), we did not observed *P. aphidis* inside the powdery mildew hypha using confocal or TEM microscopy (Figures 4, 5 and Supplementary Movies 1, 2). This suggests that *P. aphidis* is an ectoparasite of *P. xanthii*, as also demonstrated with *Trichoderma* spp. on *Pythium*, *Rhizoctonia solani*, *Sclerotium rolfsii*, and *Sclerotinia sclerotium* (Chet et al., 1981; Elad et al., 1983; Hubbard et al., 1983; Inbar et al., 1996). Although we could not observe any penetration of *P. aphidis* into the powdery mildew hyphae (Figures 4, 5 and Supplementary Movies 1, 2), we could demonstrate chitinase activity *in vitro* (Figure 6). It has been shown that attachment of an antagonist to a pathogen plays a major role in the former's biological activities, enabling cell wall degrading enzymes to be effective (Jones, 1994; Askary et al., 1997; Cook et al., 1997). The abundance of *P. aphidis* on *P. xanthii* (Figures 1, 2), its coiling around *P. xanthii* as demonstrated by SEM (Figure 3), and its chitinase activity demonstrated *in vitro* (Figure 6), suggest that *P. aphidis* parasitizes the powdery mildew hyphae. *P. aphidis* probably disrupts the pathogen's tissue via the chitinase activity and releases the nutrients required for abundant growth of *P. aphidis*. Moreover we were able to demonstrate antibiosis of *P. aphidis* crude extract against *P. xanthii in planta* (Table 2). This might contribute to its biocontrol ability by damaging and even killing powdery mildew cells. This is the first case, as far as we know, that mycoparasitism is demonstrated as a mode of action of a yeast-like biocontrol agent against powdery mildew. The literature mostly describes antibiosis as a mode of action of yeast-like biocontrol agents such as *P. flocculosa* (Bélanger et al., 1994; Hajlaoui et al., 1994; Benyagoub et al., 1996; Avis and Belanger, 2001; Avis et al., 2001; Hammami et al., 2010, 2011) and *Tilletiopsis* species (Urquhart and Punja, 1997, 2002) against powdery mildew. While mycoparasitism against powdery mildew was demonstrated as a mode of action used by biocontrol fungi such as *Veticillium lecanii* (Askary et al., 1997, 1998) and by *Ampelomyces quisiqualis* on cucumber plants (Rotem et al., 1999).

The chitinase activity of *P. aphidis* L12 (Figure 6), is supported also by recently released DNA sequence of *P. aphidis* (isolate DSM 70725) demonstrating one chitinase gene and two other candidate genes that are related to chitinase (Lorenz et al., 2014). Furthermore, the sequenced isolate contains one extracellular aspartic proteinase and eight potential glucanases (Lorenz et al., 2014); these cell wall-degrading enzymes might be involved in degradation of the pathogen cell wall, but the availability and intactness of those genes and their protein activity must be further characterized in our isolate. Furthermore, genome and transcriptome analysis of the biocontrol agent *P. flocculosa* demonstrated differences from the pathogenic related fungi *U. maydis* in genes related to fungal cell wall degradation and other gene families related to *P. flocculosa*'s epiphytic and antagonistic characteristics, such as lipases, chitinases and chitin binding proteins (Lefebvre et al., 2013).

The released sequences of the genomes of *Pseudozyma* spp. provide further support for antibiosis as they reveal the presence of gene clusters regulating synthesis of glycolipids, some of which have an antibiosis ability (Lefebvre et al., 2013; Morita et al., 2013a, 2014; Lorenz et al., 2014; Saika et al., 2014). Flocculosin, synthesized by *P. flocculosa* is an example of such glycolipid (Teichmann et al., 2007, 2011a,b). We speculate *P. aphidis* isolate L12 also has the ability to produce glycolipid/s with antibiosis ability. However, antibiosis as a mode of action *in vivo* is still questionable since *U. maydis*, which synthesize ustilagic acid, an antifungal compound very similar to flocculosin, does not have any antagonistic effect against powdery mildew (Hammami et al., 2011).

Collectively, our data show that the *P. aphidis* isolate L12 has potential for use as a biocontrol agent against powdery mildew via mycoparasitism. We also suggest antibiosis as a possible mechanism based on our observation that spore-germination of powdery mildew is inhibited *in planta* by the crude extract of metabolites of *P. aphidis*. We show morphological yeast-to-hypha transition of *P. aphidis* that parasitize the powdery mildew. However, to extend our knowledge, it is important to identify the cues causing *P. aphidis* dimorphism in association with *P. xanthii*.

## Author contributions

ML designed the experiments. AG, KB, CC, RH, and AD performed the experiments. EZ preform the confocal microscopy analysis. ML analyzed the results and wrote the manuscript. All authors read and approved the final manuscript.

### Conflict of interest statement

The authors declare that the research was conducted in the absence of any commercial or financial relationships that could be construed as a potential conflict of interest.
