# Supplementary material for: Biological control of the cucurbit powdery mildew pathogen Podosphaera xanthii by means of the epiphytic fungus Pseudozyma aphidis and parasitism as a mode of action
Source: Front Plant Sci. 2015 Mar 11;6:132. doi: 10.3389/fpls.2015.00132 (PMC4356082; doi:10.3389/fpls.2015.00132)
Supplement: Supplementary file 1 [file DataSheet1.DOCX]

***Supplementary Material***

**Biological control of the cucurbit powdery mildew pathogen *Podosphaera xanthii* by means of the epiphytic fungus *Pseudozyma aphidis* and parasitism as a mode of action**

**Aviva Gafni^1^, Claudia E. Calderon^1^, Raviv Harris^1^, Kobi Buxdorf^1^, Avis Dafa-Berger^1^, Einat Zelinger-Reichert^2^ and Maggie Levy^1*^**

^1^Department of Plant Pathology and Microbiology, The Robert H. Smith Faculty of Agriculture, Food and Environment; The Hebrew University of Jerusalem, Rehovot, Israel.

^2^Interdepartmental Equipment Facility, The Robert H. Smith Faculty of Agriculture, Food and Environment; The Hebrew University of Jerusalem, Rehovot, Israel.

*Correspondence: maggie.levy@mail.huji.ac.il; The Robert H. Smith Faculty of Agriculture, Food and Environment, Herzel st. Lauterman Build. P.O. Box 12, Rehovot 76100, Israel; Tel.: +972-8-948-9162; Fax: +972-8-946-6794

1. **Supplementary Material and Methods**

***P. aphidis* transformation**

*P. aphidis* transformation was carried out as previously reported by Morita et al. ([Morita et al., 2007](#_ENREF_38)), with some modifications as we describe below. *P. aphidis* was grown in PDB at 25°C and agitated at 150 rpm for 4 to 7 days. The cells were collected by centrifugation at 4000 *g* and the pellet was washed in ice cold water. The washing steps were repeated five times and the pellet was finally suspended in ice cold 1 M sorbitol and the cells were diluted in 1 M sorbitol to 1 × 10^9^ cells/ml. Fifty-μl aliquots were then electroporated with 5 μl of 100 ng/ml plasmid pOTEF-SG DNA carrying the hygromycin B (Hyg) selection cassette and a GFP-encoding gene under the control of the *Ustilago maydis* *tef* promoter ([Spellig et al., 1996](#_ENREF_43)) with electrodes separated by 0.2 em and the voltage set to 1.5 kV. The electroporated cells were immediately diluted in 1 ml of ice cold PDB and an aliquot of 0.1 ml was then spread onto a PDA plate containing 100 μg/ml Hyg. Putative transformants were re-plated onto fresh selection plates three times at 2-3 day intervals. Putative transformants were subjected to colony PCR using Hyg and GFP primers (**Supplementary Figure 1A**) and GFP expression was verified by fluorescence microscopy (**Supplementary Figure 1B**). The GFP mutant used in this study demonstrated the same growth rate and morphology as the wild type strain. The following primers were used for the colony PCR analysis: Hyg-F 3'ACCCATATGAAAAAGCCTGAACTC; Hyg-R 3'GCATATGAAATCACGCCATG; GFP-F 3'ACTGGGTGCTCAGGTAGTGGTTGT and GFP-R 3'AAGCTGACCCTGAAGTTCATCTGC.

**Colonization of leaves by *P. aphidis***

*P. aphidis* strain L12 was grown in potato dextrose broth (PDB) at 25°C and agitated at 150 rpm in the dark for 4 to 10 days. The cells were collected by centrifugation at 4000*g* and the pellet was suspended in sterile deionized water to the required final concentration after counting the cells in a hemocytometer. A suspension of *P. aphidis* strain L12 (10^8^ colony-forming units [CFU]/ml in sterile deionized water) was sprayed onto 18-day-old cucumber seedlings to run-off. Treated plants were maintained in a controlled-environment chamber (25°C and 90% relative humidity) and CFU were counted on 0, 3, 6, 9, 12, 15, 18 and 21 days after treatment. At each time point, 6 cotyledons were collected randomly from 6 different plants. Cotyledons were weighed and then homogenized in sterile deionized water. The suspensions were serially diluted, transferred onto PDA plates and incubated at 25°C for 2 days. Colonies were then counted and data were expressed as log CFU/mg FW. The experiment was repeated two times with similar results.

***in vitro* dimorphism assays**

For *in vitro* dimorphism assays, *P. aphidis* was also grown in yeast malt peptone dextrose (YMPD: yeast extract, 3 g/liter; malt extract, 3 g/liter; peptone, 5 g/liter; dextrose, 10 g/liter) and 1 X Murashige and Skoog (MS) salt mixture including vitamins (Duchefa Biochemie, Netherlands) supplemented with 1% (w/v) sucrose (Murashige and Skoog, 1962).

1. **Supplementary Figures**


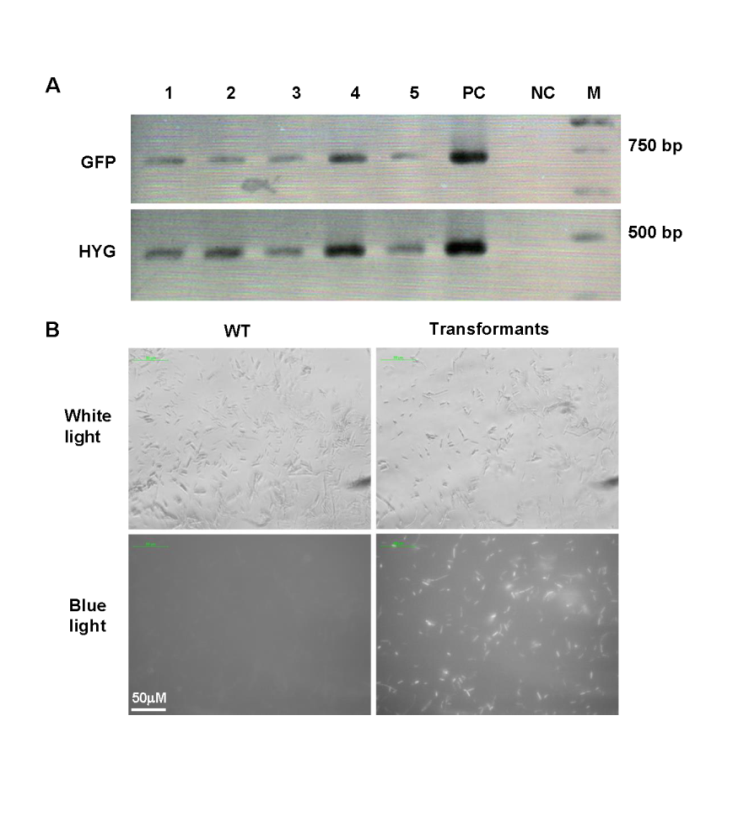


**Supplementary Figure 1. Transformation of *P. aphidis*.** (A) PCR analysis of several transformants (lanes 1-5) for the presence of hygromycin resistance (HYG) and green fluorescence protein (GFP) -encoding genes. (B) Fluorescence microscopy analysis of the selected transgenic *P. aphidis* isolate (L12) for GFP expression. M, molecular markers; PC, positive control (vector); NC, negative control (wild type *P. aphidis* isolate L12).

**Supplementary Figure 2.** **Population dynamics of *P. aphidis* on cucumber plants.** Quantification of *P. aphidis* on treated leaves over 21 days expressed as log_10_ of the number of colony-forming units (CFU) per gram(gr) fresh weight (FW) of leaf tissue (log_10_CFU/gr FW). Shown is one representative experiment out of at least three more experiments with similar results.

**Supplementary Figure 3.** Powdery mildew coverage on cucumber leaves. Plants treated with *P. aphidis* (PA) or water, as control (Control), were inoculated 3 days post treatment with *P. xanthi* . Powdery mildew coverage is illustrated 16 days post inoculation.

**Supplementary Figure 4. Dimorphism of *P. aphidis* on different media.** *P. aphidis* demonstrated different morphism when grown on YMPD, MS or PDA media for 5 days at 25^o^C under light or dark conditions.


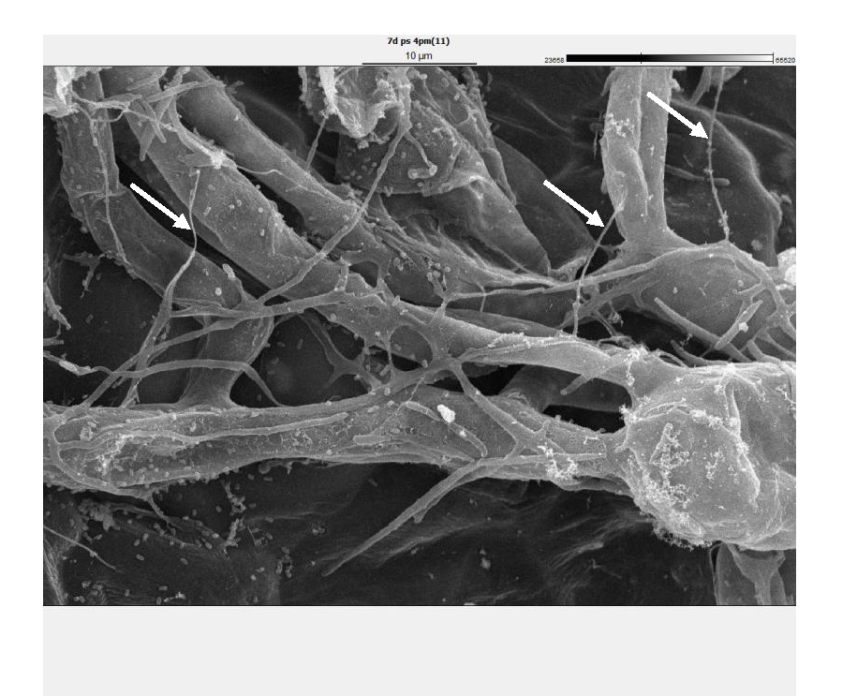


**Supplementary Figure 5.** ***P. aphidis*–powdery mildew interactions on cucumber cotyledons.** SEM microscopy of cucumber cotyledons treated with *P. aphidis* and infected with *Podosphaera xanthii*. *P. aphidis* thin hyphae extending from one powdery mildew hypha to another one are indicated with white arrows.

**Supplementary Figure 6. Antibiosis effect on *P. xanthii* spore germination.** Cucumber cotyledons disks were treated with *P. aphidis* crude extract, distilled water or ethyl acetate as controls. The percentage of conidia germination was evaluated by bright field microscopy 24 hours after inoculation. Representative pictures showing the effect of each treatment are shown. (A) disks treated with ethyl acetate. (B) disks treated with distilled water. (C) disks treated with *P. aphidis* crude extract. The left corner of each panel demonstrates higher magnification. The size bars represent 40μM in all panels. Red arrows indicate hypha and black arrows indicate germination tube.

1. **Supplementary Tables**

**Supplementary Table 1.**  Effect of *P. aphidis*

treatment on *P. xanthii* germination *in planta*

| ***P. xanthii* germination tube length 2 days post-inoculation (cm)** | |
| --- | --- |
| Control | PA |
| 6.50± 0.58 | 4.75± 0.20^*^ |

Value followed by asterisk

is significantly different from control according

to Student's *t*-test; *P=*0.016; n=6.

1. **Supplementary Movies**

**Supplementary Movie 1. Ectoparasitism demonstrated by Confocal 3D video**. *P. aphidis* hypha (green) coiled around powdery mildew hypha (red) and cross sectioning images demonstrating this interaction.

**Supplementary Movie 2. Ectoparasitism demonstrated by Confocal 3D video.** *P. aphidis* hypha (green) coiled around powdery mildew hypha (red) and cross sectioning images demonstrating this interaction.

1. **References**

Morita, T., Habe, H., Fukuoka, T., Imura, T., and Kitamoto, D. (2007). Convenient

transformation of anamorphic *basidiomycetous* yeasts belonging to genus *pseudozyma* induced by electroporation. *J. Biosci. Bioeng*. 104, 517-520.

Murashige, T., and Skoog, F. (1962). A revised medium for rapid growth and bioassays with tobacco tissue cultures. *Physiol. Plant*. 15, 473-497.

Spellig, T., Bottin, A., and Kahmann, R. (1996). Green fluorescent protein (GFP) as a new vital marker in the phytopathogenic fungus *Ustilago maydis*. *Mol. Gen. Genet*. 252, 503-509.
